# Supplementary material for: From Water to Land: The Structural Construction and Molecular Switches in Lungs during Metamorphosis of Microhyla fissipes
Source: Biology (Basel). 2022 Mar 30;11(4):528. doi: 10.3390/biology11040528 (PMC9030589; doi:10.3390/biology11040528)
Supplement: Supplementary file 1 [file biology-11-00528-s001.zip › biology-1582553-supplementary.pdf]

Table S1. Developmental phase information of each sample.

Sample	Pro-metamorphosis	Metamorphosis Climax	Post-metamorphosis	Adult
L1_1	1	0	0	0
L1_2	1	0	0	0
L1_3	1	0	0	0
L2_1	2	0	0	0
L2_2	2	0	0	0
L2_3	2	0	0	0
L3_1	3	0	0	0
L3_2	3	0	0	0
L3_3	3	0	0	0
L4_1	0	1	0	0
L4_2	0	1	0	0
L4_3	0	1	0	0
L5_1	0	0	1	0
L5_2	0	0	1	0
L5_3	0	0	1	0
L6_1	0	0	0	1
L6_2	0	0	0	1
L6_3	0	0	0	1
